# Nutritional Requirements During Pregnancy and Lactation: An Editorial

**DOI:** 10.3390/nu18040662

**Published:** 2026-02-18

**Authors:** Antonios Siargkas, Panagiota Kripouri, Antigoni Tranidou, Themistoklis Dagklis, Ioannis Tsakiridis

**Affiliations:** Third Department of Obstetrics and Gynaecology, School of Medicine, Faculty of Health Sciences, Aristotle University of Thessaloniki, 54124 Thessaloniki, Greece

## 1. Introduction

As we navigate the landscape of nutritional science in 2026, global efforts remain steadfast in their commitment to optimizing maternal and offspring health, which was reinforced at the 78th World Health Assembly in Geneva, where the World Health Organization (WHO) reaffirmed its global nutrition targets, placing renewed emphasis on maternal dietary intake. Crucially, the Assembly expanded upon pre-existing goals by explicitly incorporating targets to combat childhood obesity and promote exclusive breastfeeding [1].

This expanded mandate reflects a critical paradigm shift, moving beyond the classical focus on resource scarcity to address the “double burden of malnutrition.” While being central to current discourse, this concept was introduced as early as 1992 at the first International Conference on Nutrition. In 1993, the World Health Assembly endorsed the World Declaration and Plan of Action, calling on members to support breastfeeding and eliminate micronutrient deficiencies [2]. Twenty-two years later, The Lancet dedicated a series of research papers to the double burden of malnutrition, highlighting its economic impact and links with obesity, undernutrition, and climate change [3]. In this series, Wells et al. suggested that major socioeconomic shifts are required to address the aetiologic pathways of this double burden [4]. These foundational works have informed the current literature landscape and the need to give effect to global resolutions.

This Special Issue, titled Nutritional Requirements of Pregnant and Lactating Women, aligns directly with the strategic axes established by the World Health Assembly, namely, the pre-conceptional, antenatal, and early offspring nutritional stages. Consequently, in this Issue we do not view malnutrition traditionally as merely describing low caloric intake but also incorporate research on adipogenic nutritional habits.

Nutrition has resurfaced in 2026 at the forefront of political discourse while reasserting itself as a subject for scientific research [5]. Despite efforts to understand the role nutrition plays in fetal and neonatal development, specific mechanisms remain complex and of continued interest. This context underscores the need for researchers to focus on evidence rather than impressions. We aspire to contribute to this scope through this Issue by shedding light on the hidden specifics behind the impact of nutrients on maternal and fetal health.

In accordance with global directives, this Special Issue hosts a diverse collection of research investigating the whole spectrum of nutritional challenges, from obesogenic diets to persistent undernutrition. The included works span various methodologies, incorporating human subjects, experimental animal models, meta-analyses, and advanced metabolomics. By integrating data from different countries and diverse perspectives, nutrition is examined through a broader lens. Covering the pre-conceptual period, pregnancy, postpartum, and the neonatal phase, these selected papers offer a comprehensive understanding of how nutrition shapes the health trajectories of mothers and their offspring.

## 2. An Overview of the Published Articles

In this Special Issue, we integrate research papers on maternal nutrition and neonatal outcomes from a multidisciplinary perspective.

To address micronutrient requirements, a systematic review was conducted on iodine intake and iodine status based on biomarkers during pregnancy and lactation (contribution 1). This analysis underscored that iodine deficiency remains a significant concern even in industrialized countries (contribution 1), and demonstrated a link between dairy product intake and iodine adequacy, identifying dairy as a vital “safety net” for preventing deficiency in populations where iodized salt usage may be inconsistent (contribution 1).

Researchers from the University at Buffalo investigated the association between maternal egg consumption and the establishment of lactation in a cohort of 1039 mother–infant dyads (contribution 2), categorizing consumption by egg components (whole vs. white) and cooking methods. The findings were robust: mothers who consumed eggs three or more times per week had significantly higher odds of initiating breastfeeding (OR 3.34; 95% CI 1.51–7.39) compared to non-consumers. The authors concluded that whole egg ingestion, specifically the nutrient-dense yolk, is linked to superior lactation outcomes.

Marine food sources are also featured in a research paper that stratified fish consumption by profession, educational level, and family size in two cohorts of pregnant women studied ten years apart (2013 vs. 2023) (contribution 4). The results revealed a positive longitudinal shift, with a significant increase in the frequency of frozen fish consumption among working women, potentially reflecting improved maternal awareness regarding the benefits of omega-3 fatty acids for fetal development (contribution 3).

The dynamic nature of breast milk was explored in an observational cohort study from Italy, which assessed the lipid profile of human milk according to the lactation semester and maternal dietary habits (contribution 4). This work supported the hypothesis that milk is not a static fluid but a dynamic biological system, adjusting its fatty acid composition to meet an infant’s developmental needs (contribution 4).

Three papers focused on the significance of Vitamin D, a highly debated nutrient, and the consequences of its deficiency. The first, a pilot study of 30 pregnant women, reported no significant correlation between cortisol stress markers (AUCg) and Vitamin D sufficiency (*p* = 0.57), despite a high prevalence of deficiency (40%) (contribution 5). The second study (n = 30) investigated the relationship between prematurity and hypovitaminosis D, validating recruitment methodologies and confirming high deficiency rates (73%) (contribution 6). The third study reviewed the literature on maternal Vitamin D and offspring neurodevelopment (contribution 8). Through analyzing data from over 18,000 mother–child pairs across 20 studies, this meta-analysis found only a weak, non-significant trend connecting maternal levels to cognitive development, underscoring the need for future research (contribution 7).

As previously noted, obesity is considered a critical form of malnutrition. In this Special Issue, we host research regarding hepatic metabolism and oxidative stress in female Muridae (rats) (contribution 8). This study provided mechanistic insight, demonstrating that a short-term obesogenic diet during pregnancy and lactation led to significant hepatic steatosis, a reduction in antioxidant capacity (lower GSH/GSSG ratio), and elevated pro-inflammatory cytokines (TNF-α). These findings validated this animal model for future foodomics research into the metabolic costs of poor maternal nutrition (contribution 8). In the human research counterpart, a systematic review found that “Westernized” dietary patterns significantly contribute to the risk of Gestational Diabetes Mellitus (GDM), whereas “Prudent” or plant-based patterns may offer protective effects, emphasizing the metabolic perils of low-quality diets during gestation (contribution 9).

Finally, in this Issue, we present two studies that enhance our global understanding of the behavioral and structural determinants of nutrition. Researchers studied lactation attitudes among university students in Hungary and Syria (contribution 10), concluding that while strong agreement exists regarding the nutritional benefits of breastfeeding, cultural background significantly influences specific attitudes (contribution 10). Additionally, a mapping review is hosted exploring the factors that contribute to maternal nutritional habits in Ethiopia (contribution 11). By integrating a socio-ecological model with the UNICEF framework, the authors identified critical gaps in terms of “care for women” and structural barriers, such as food insecurity and lack of education, that dictated nutritional outcomes (contribution 11).

## 3. Conclusions

Nutritional science represents a shifting landscape; humanity’s understanding has evolved from a simplistic view of malnutrition as merely a caloric deficit to complex modern concepts such as the “double burden of malnutrition” and specific micronutrient deficiencies. Contemporary research now integrates advanced data analytics and metabolomics to provide clinical medicine with a clearer picture of how high-quality nutrition helps to maintain physiological homeostasis. These advancements carry profound implications for determining the necessary practical changes in both clinical protocols and the global health status quo.

This paradigm shift is particularly relevant during early human development, a period characterized by significant biological plasticity. Current evidence suggests that nutritional interventions can modulate biochemical pathways and influence genetic and epigenetic mechanisms, ultimately affecting the number of disease-free years an individual will experience. Within this framework, further investigation is required to elucidate precisely how nutrition in utero and during early life impacts these long-term trajectories. Such research is essential for translating theoretical models into current knowledge, providing the springboard for major public health interventions. The authors hope that this Special Issue will contribute meaningfully toward this vital goal.
